# The semi-automated development of plant cell wall finite element models

**DOI:** 10.1186/s13007-023-00979-2

**Published:** 2023-01-09

**Authors:** Andrew Sayad, Yusuf Oduntan, Norbert Bokros, Seth DeBolt, Alice Benzecry, Daniel J. Robertson, Christopher J. Stubbs

**Affiliations:** 1 School of Computer Sciences and Engineering, Fairleigh Dickinson University, Teaneck, NJ USA; 2 Department of Mechanical Engineering, University of Idaho, Moscow, ID USA; 3 Department of Horticulture, University of Kentucky, Lexington, KY 40546 USA; 4 Department of Biological Sciences, Fairleigh Dickinson University, Teaneck, NJ USA

## Abstract

This study presents a methodology for a high-throughput digitization and quantification process of plant cell walls characterization, including the automated development of two-dimensional finite element models. Custom algorithms based on machine learning can also analyze the cellular microstructure for phenotypes such as cell size, cell wall curvature, and cell wall orientation. To demonstrate the utility of these models, a series of compound microscope images of both herbaceous and woody representatives were observed and processed. In addition, parametric analyses were performed on the resulting finite element models. Sensitivity analyses of the structural stiffness of the resulting tissue based on the cell wall elastic modulus and the cell wall thickness; demonstrated that the cell wall thickness has a three-fold larger impact of tissue stiffness than cell wall elastic modulus.

**Supplementary Information:**

The online version contains supplementary material available at 10.1186/s13007-023-00979-2.

## Introduction

Stalk lodging is a known problem affecting the agriculture industry and results in billions of dollars every year in lost yield [1, 2]. Stalk lodging occurs when externally applied forces exceed the maximum load that the stalks are able to withstand. The mechanical failure of plant stalks is a multiscale phenomena that starts at the cellular level, and propagates out to the tissue, organ, and ultimately the whole plant scale [3]. It is thus important to understand and quantify the stresses induced in cell walls during stalk bending. It is likewise important to understand and quantify microstructural phenotypes that directly affect the mechanical response of plant tissues. Development of mechanistic and first principle models can be used to achieve these objectives by simulating the cellular environment and the forces it experiences [4]. In particular, Finite Element Models (FEMs), which are a powerful tool used in engineering analyses, can be utilized to create physically realistic digital environments, in which cellular structures can be tested in silico. Such models have previously been developed at the plant [5], organ [6, 7], and tissue level previously [8]. However, the creation of cellular level models requires digitization of cell images is traditionally done through time-consuming and fairly expensive methods [6, 9, 10]. The purpose of this study is therefore to present a relatively inexpensive method for high-throughput digitization and quantification of plant cells that can be used to create two-dimensional FEMs. These FEMs can in turn be used to analyze and correlate mechanical stresses in cellular structures with that of cell wall morphology and integrity. Further, this tool will allow investigation of the effects of cellular organization and morphology on mechanical tissue properties.

Current methods of cell imaging include mass spectrometry, fluorescence imaging, vibrational microspectrometry, and x-ray computed microtomography [11]. All of these methods utilize intense sample preparation and highly specialized equipment. Mass spectrometry is a very common and effective cell imaging technique but requires strict sample preparation control. Along with this, it has been documented that the imaging process is far from routine, and the specimen preparation is sample-dependent [12]. These challenges make it difficult to streamline the mass spectrometry process. Another method used is fluorescence imaging. This technique requires an intense sample preparation process that involves drying samples for 24 h and carefully choosing mounting media as it affects the fluorescence. This method is also frequently used on cross sections (slices of the stem) as well as particles (ground into a powder). In order for clear results, the mounting media and preparation technique must be kept the same for both [13]. This adds complexity to the imaging process and increases the overall time it takes to obtain the images. X-ray Microtomography and Raman vibrational microspectroscopy are other methods also used for cell imaging. They both require expensive specialized equipment to obtain the images. Documentation for Raman microspectroscopy shows that samples are prepared by being freeze dried, cut with a vibratome, and soaked in acetone. Along with this, microscope calibration is also involved [14]. Microtomography involves soaking the samples overnight, cutting with a vibratome, and staining the samples with precise chemical proportions [15]. Development of a more economical imaging and digitization methodology would enable numerous research labs to develop FEMs of plants and begin to investigate several phenomena in the realm of plant biomechanics.

Box 1 Definitions

**Table:** 

WEKA	Waikato Environment for Knowledge Analysis [16, 17]
AI	Artificial Intelligence; any system that perceives its environment and takes actions that maximize its chance of achieving its goals [18]
FEM	Finite Element Method; general numerical method for solving partial differential equations in two or three space variables [19]
ML	Machine Learning; computer algorithms that can improve automatically through experience and by the use of data [20]
GUI	Graphical User Interface; user interface that allows users to interact with electronic devices through graphical icons [21]
Micro-CT (μCT) Scan	Microtomography; X-rays to create cross-sections of a physical object that can be used to recreate a virtual model [22]
Euclidean Distance Map (EDM)	indicates, for each pixel in the objects of the originally binary picture, the shortest distance to the nearest pixel in the background (or the objects) [23]

## Methods

To test the robustness of the digitization and quantification processes, samples were taken from various plants, *Zea mays* (maize), *Arabidopsis thaliana, Mussatia hyacinthine, Arrabidacea verugosa*, and submitted to several sectioning methodologies presented in the Discussion section [24]. For this study, the herbaceous representatives were manually stressed while the woody samples were naturally stressed. To bound the experiment, the primary specimens used in this study were maize specimens that were sectioned and stained as described in [6]. These were chosen to represent a “worst-case scenario”, as it is a high-throughput but relatively crude cross-sectioning and staining procedure.

### Plant materials

#### Sample acquisition, selection and preparation for microscopy

##### *Zea mays*

Experimental stalks were collected from border rows within a larger experiment located on the University of Kentucky Spindletop Research Farm. Seed was sourced from a single bag of Pioneer P1464AML (mid-season, 114 days to mature), hand planted on 05/14/2021 and fully mature stalks were harvested around 09/09/2021, approximately 118 days after planting. Immediately before planting, the field was rototilled and a precision planter was used to pre-cut 2-inch deep furrows with 30-inch row-to-row spacing. Planting density was set to 3 seeds/ft within border rows or approximately 4700 plants/acre. Weeds were controlled with a single 2.5 qt/acre treatment of Acuron supplemented with 150 lb/acre of nitrogen applied on 06/01/2021. After herbicidal activity wore off, weeds were controlled manually with hoeing as needed; neither supplemental irrigation nor pesticide treatments were required during the growing season. During stalk collection, individuals were cut from the ground at their base using garden shears and at the internode above the primary ear bearing node. All leaves, and leaf sheaths were removed from individual stalks. Stalks were spread in a single layer on wire rack benchtops in a greenhouse set to 36 °C w/adequate air circulation to deter mold growth and allowed to dry for one month. After drying, stalks were cut into subsections of approximately 3 internodes before shipment to Fairleigh Dickinson University, where they were stored at standard office temperature and humidity.

The samples were sectioned using a 110 V, 6 in. trim saw with a thin-notched diamond saw blade. The samples were cut in such a way that they maintained structural rigidity, in that they were able to be gently handled without damaging the specimen. They were also cut to be thin in order to allow light through to enhance the images captured by the microscope. The samples were then stained with Safranin O for 15–30 s. They were then left to dry for 1–2 min before being placed under the microscope for image capturing.

##### Arabidopsis thaliana

Samples were germinated and grown to 6 weeks of age in soil-less media under a 16:8 light dark regime. Stem samples were obtained by plant sacrifice and sections prepared from the lowest internode on the mature stem. These samples were prepared by thinly slicing transverse sections of the stem with single edged razor blades (VWR, 76457-428 Radnor, PA, USA) supported against a firm background of a longitudinal halves carrot. Once sliced, they were counterstained within an aqueous toluidine blue solution for 30 s, washed in water and imaged.

##### Mussatia hyacinthine and* Arrabidacea verugosa*

Mature stem samples were collected on tropical forests of Central America. Samples were processed and prepared for microscopy following traditional protocols as described on [24].

### Microscopy

**Fig. 1:**
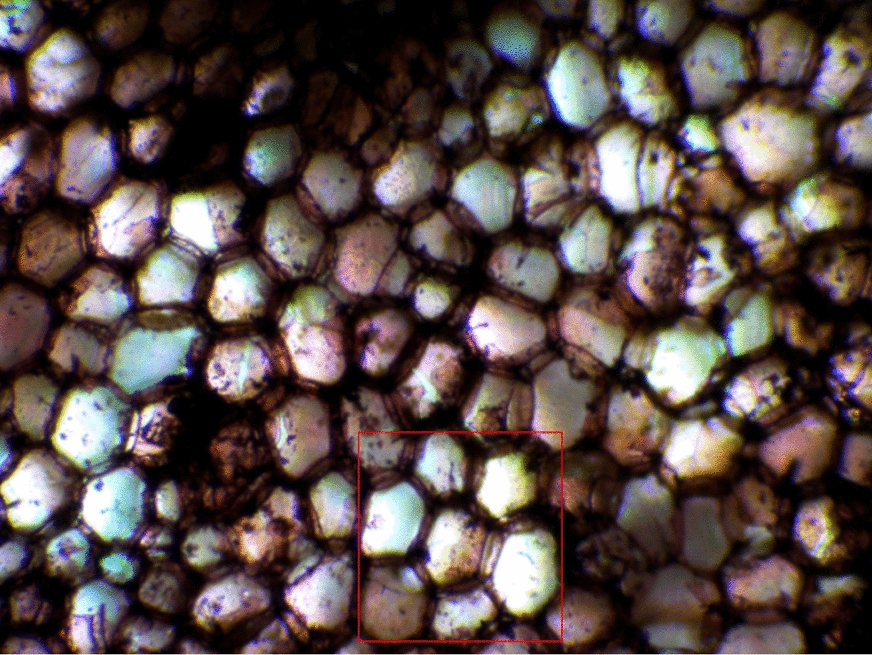
Original microscope image of *Zea mays* stem cross-section (100 ×) with red square highlighting the cropped region

The microscope used to view the samples was the AmScope LED Binocular Compound Microscope with a 5 megapixel USB3.0 camera and the software used to capture the images was AmScope 2021 v4.11. Samples were selected based on the clarity of the cells after viewing under the microscope. After cutting the samples, many cells tend to rip or tear causing them to overlap, which then causes problems during the image processing. To minimize this, samples were chosen to have clear cell boundaries and a high contrast between cell walls and inner cells. All samples were view at a magnification of 100X. The images were then cropped using the same selection criteria (Fig. 1).

#### Image processing

The image is cropped (highlighted by a red rectangle) and processed using the functions in the open source image processing software, FIJI/ImageJ. The image is first converted to an 8-bit image. It is then sharpened and the brightness and contrast were adjusted to a minimum value of approximately − 10 and a maximum value of 127. These values vary slightly between each image, the goal of this step is to increase the contrast between the cell walls (dark) and the inner cell (bright) in preparation for the machine learning segmentation; the greater the contrast is, the more effective the segmentation will be. The despeckle function is used three times to clean the image and remove the background noise created from cutting the sample. After the image is clear with high contrast, WEKA (Waikato Environment for Knowledge Analysis) machine learning segmentation is conducted; see the Additional file 1 for more details. The inner cells and the cell walls are categorized into two different classes based on user selections.

This image was processed in a similar fashion as the previous image. It was cropped, converted to 8-bit, and was sharpened and despeckled according to user judgment. The difference in processing for this image was that the brightness and contrast was adjusted to a minimum value of 20 and a maximum value of 112. During the WEKA segmentation stage of the processing, the FIJI magic wand tool was used for making some selections. The magic wand tool automatically highlights regions and boundaries on the image by clicking instead of requiring the user to manually outline the boundaries. The magic wand tool works well for cells that already have clear boundaries. The manual selections must still be made in conjunction with the magic wand tool in order to achieve the diversity that is required for a clear WEKA segmentation.

### Manual thresholding: alternative to WEKA machine learning

**Fig. 2:**
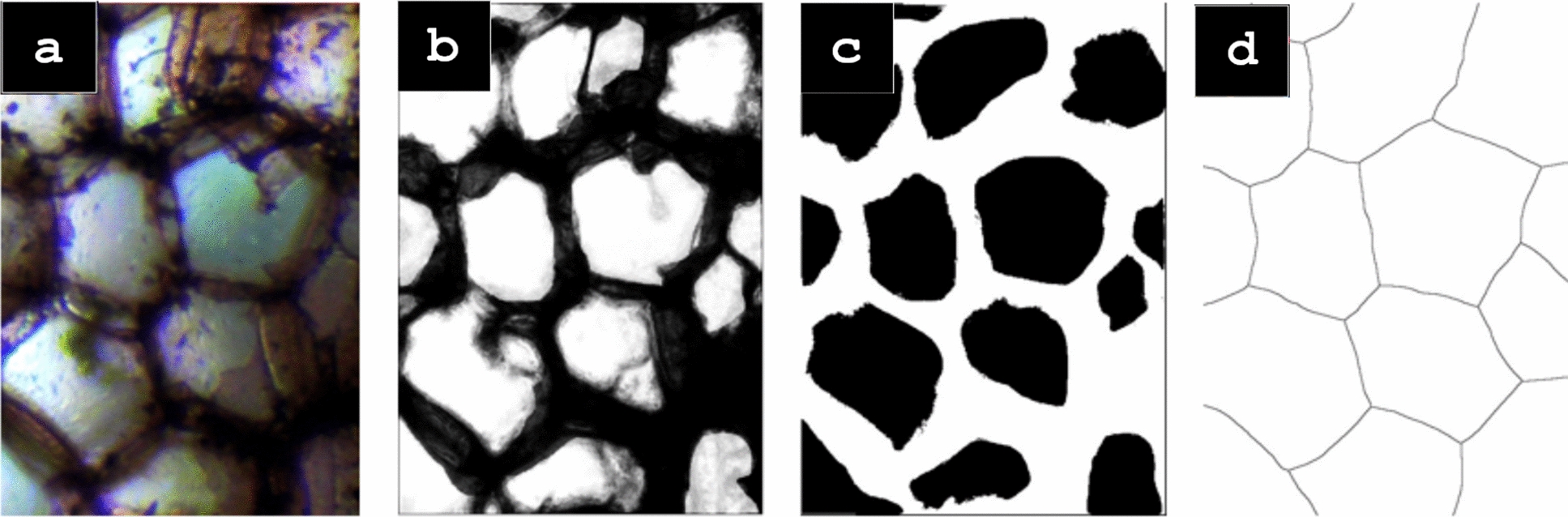
*Zea mays* stem cross-section (100 ×) (from left to right) cropped microscope image (**a**), output of WEKA (**b**), binarized and cleaned image (**c**), Voronoi splines (**d**)

**Fig. 3:**
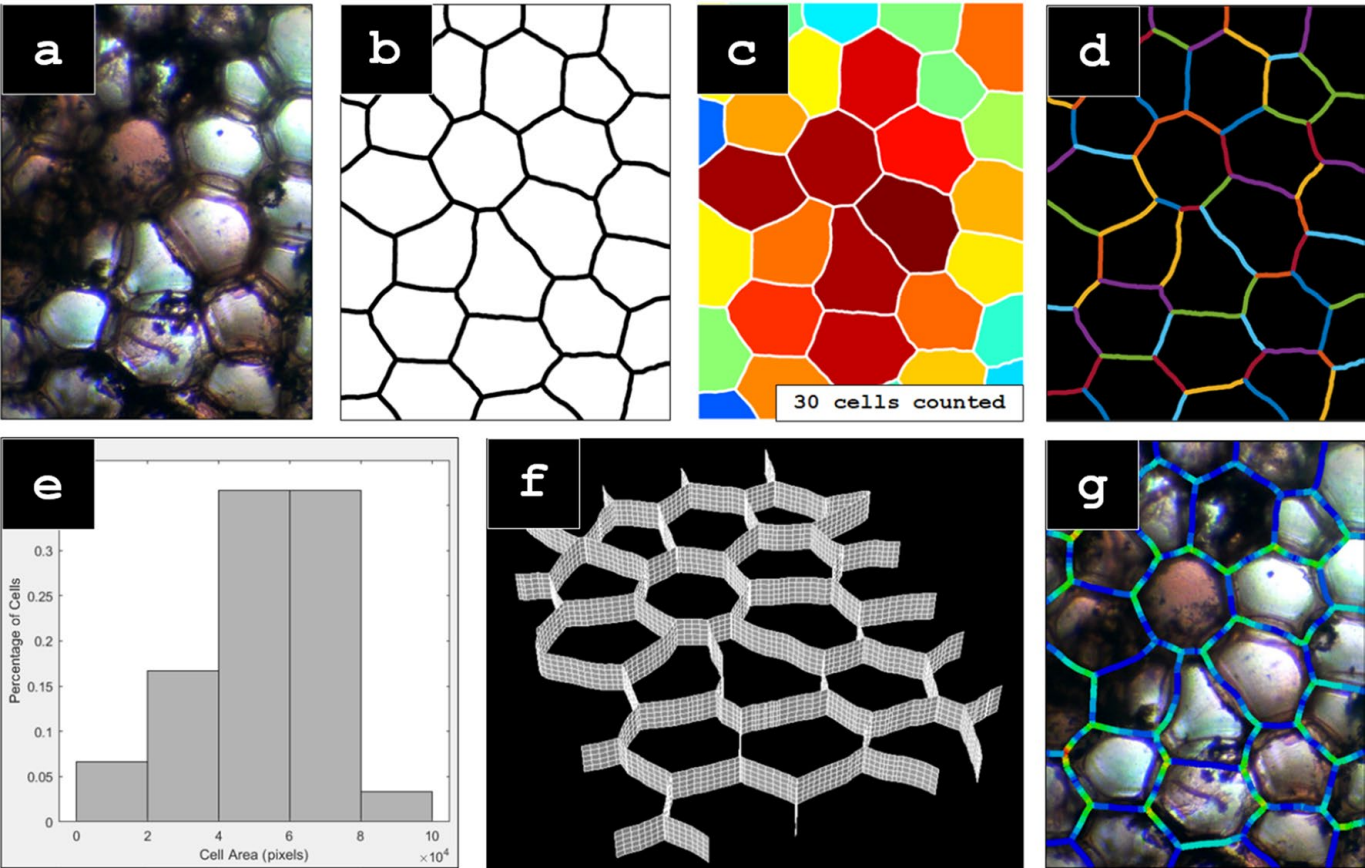
An overview of the digitization and modeling process, including some of the output functions: (**a**) a compound microscope image of the cellular structure of a stained specimen of *Zea mays* stem cross-section (100 ×); **b** the Voronoi curves output from the ImageJ post-processing workflow; **c** the output of ColorCellsBySize, which colors each cell from smallest (blue) to largest (red), and counts the total number of cells, **d** the output of CreateFEM, showing the resulting splines converted into Python script for importing into Abaqus/CAE, (**e**) the output of MeasureCellSize, showing a histogram of the cell sizes normalized to the total quantity of cells, (**f**) a 3-dimensional rendering of the FEM developed from the output splines, (**g**) a stress analysis of the structure, depicting von Mises stress from minimum (blue) to maximum (red) stresses, overlaid onto the original compound microscope image

**Fig. 4:**
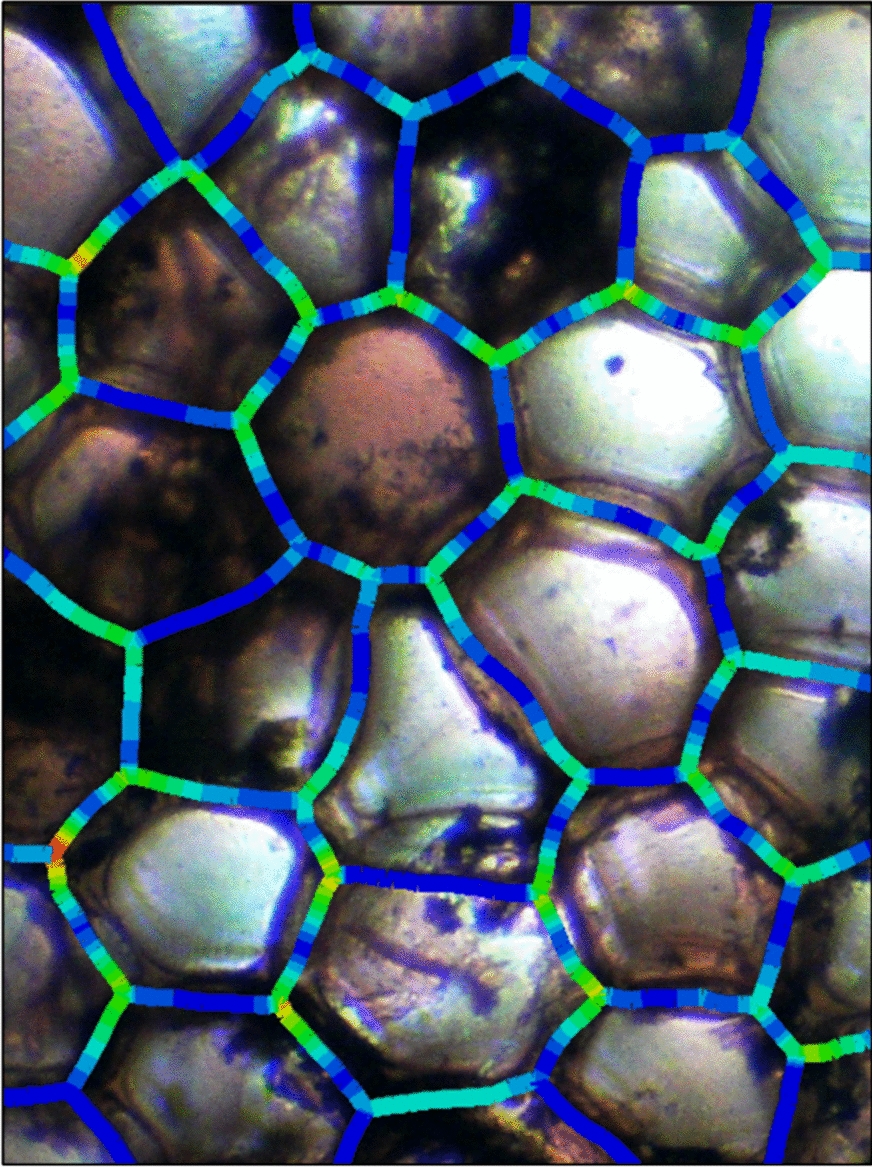
**a** *Mussatia hyacinthine* stem cross-section (100 ×) with cropped region highlighted by the rectangle; (**b**) the resulting Voronoi splines

**Fig. 5:**
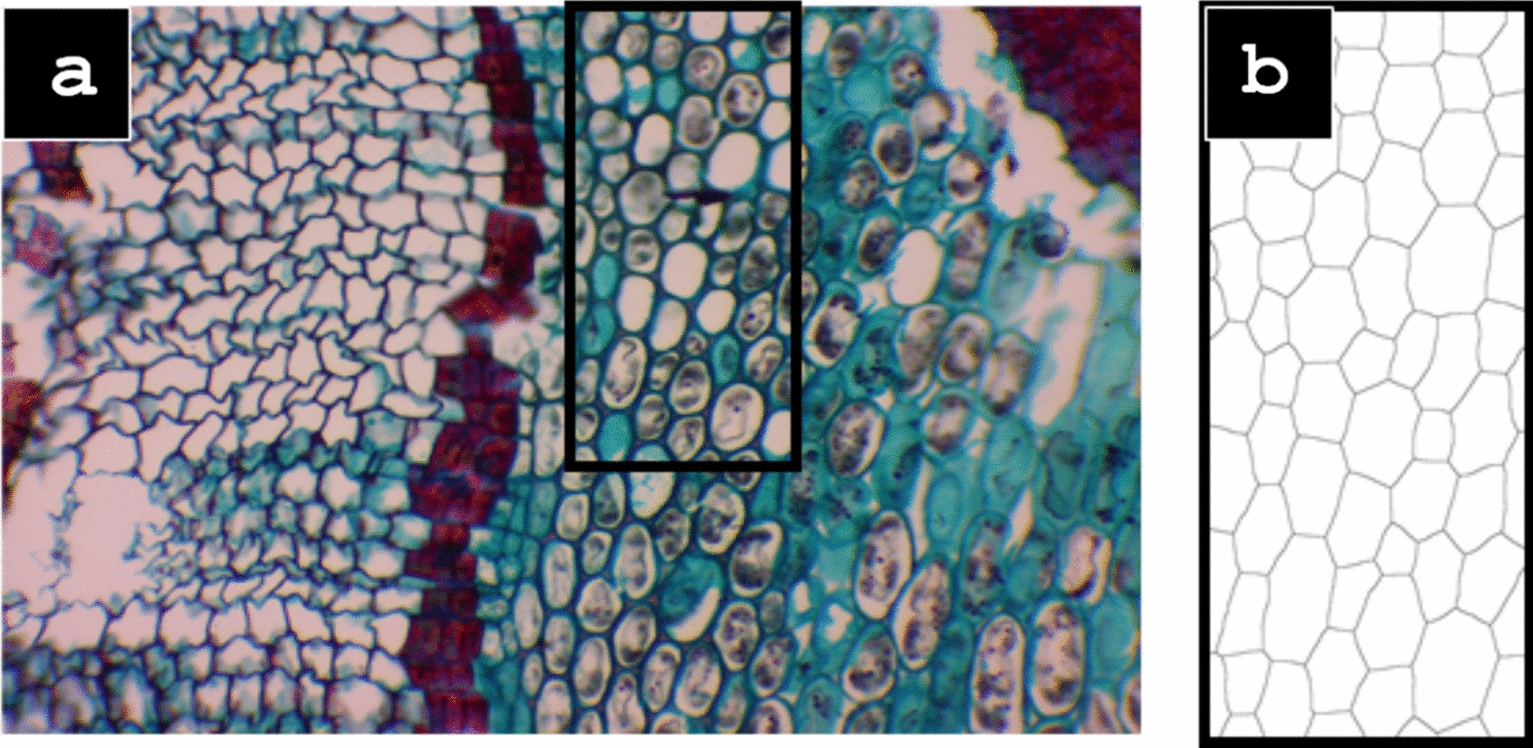
**a**, **c** *Arabidopsis thaliana* stem cross-section (100 ×) with cropped region highlighted by the rectangle; (**b**, **d**) the resulting Voronoi splines

As an alternative to the WEKA segmentation method, a more labor-intensive and semi-automated method was developed in which manual thresholding was utilized as opposed to the machine learning thresholding. The images were binarized and the threshold was adjusted. This first thresholding was done with large tolerances where all of the cells in the image were present while also limiting noise. The typical range for the first threshold was 0 for the minimum and around 175 for the maximum. The remaining noise and irregularities leftover from the threshold were then eliminated using FIJIs freehand selection, delete, and invert tools. Limiting the noise and interference greatly improves the software’s segmentation capabilities. The image was then converted into a Euclidean Distance Map (EDM), in which the points of the image are assigned a value equal to the largest radial distance that fits into the binary point [25]. The next step was a second binarization and thresholding where the boundaries of the cells were maintained while also being made as thin as possible without the cells colliding into each other. This step is left to user judgment and may take several attempts to find the best thresholding values until a user intuition is developed (Figures 2, 3, 4, 5).

### Voronoi splines

In order to define the cell wall boundaries, Voronoi splines were created using FIJI. A Voronoi diagram is a partitioning of a plane into regions [26]. The regions are formed about a given set of points called Ultimate Eroded Points (UEPs) that are generated by FIJI’s distance map function. The FIJI Voronoi function creates splines of points being equidistant to the borders of the two nearest points [25]. The cell wall splines were developed after the pre-processing and segmentation of the images detailed in the previous section. The FIJI Voronoi function is then used on the image, creating cell wall splines that retain the overall layout of the original image. Thresholding was done one final time on the image to ensure that all of the splines are clearly visible, this is also left to user judgment although it is a much easier process than the first thresholding. The final step was to ensure that the Voronoi function did not create any irregularities, usually showing up as a triangular shape branching off a cell wall. To correct these, they were simply selected with the freehand selection tool and deleted. See Fig. 2 comparing stem cross-section cropped microscope image (a), output of WEKA (b), binarized and cleaned image (c), Voronoi splines (d).

### Matlab

The Voronoi splines are then processed in Matlab using a set of custom functions; see the Additional file 1. The function *ColorByCellSize* counts the number of cells in the image, and colors each cell from blue (small) to red (large) based on the size of the cell. This allows researchers to quickly quantify the number of cells, and get a visual representation of the distribution of cell sizes throughout the image. For a more quantifiable cell size measure, *MeasureCellSize* produces a histogram of the sizes of the cells in the image. Finally, *CreateFEM* traces the Voronoi splines and creates a Python Script file (*.py) that can be executed in Abaqus/CAE to create a two-dimensional FEM of the cell wall structure. These functions are available at the github repository https://github.com/cstubbsFDU/PlantCellWallFEM. An overview of the digitization and modeling process, including some of the output functions can be seen on Fig. 3

### Finite element model

The finite element models were developed in Abaqus/CAE 2021. Each FEM was built as a two-dimensional model, using the geometry output from the Matlab function *CreateFEM*. The model was then converted from pixels to microns using the microscope resolution of the images. The geometry was meshed using two-node linear beam elements (B21) with a seed size of 1 μm. The cell walls were modeled as a uniform thickness of 4.3 μm for the *Zea mays* samples, as discussed in the next section. The model was two-dimensional with an assumption of plane stress. A single homogeneous material was used for the cell walls, which was assumed to be linear-elastic and isotropic. The cells were assumed to be hollow, with no cell interior material modeled. Many of these assumptions are admittedly simplifications of the cellular microstructure, and are discussed in further detail in the Limitations section. Boundary conditions fixed the bottom nodes in the vertical direction, fixed the leftmost and rightmost nodes in the horizontal direction, and stretched the top nodes by 1 μm. The FEMs were analyzed using a non-linear, full Newton direct solver in Abaqus/Standard 2021. From the resulting output database, the reaction force was extracted from the model to obtain a stiffness value, and the maximum stress in the cell walls were queried.

### Cell wall thickness

Unfortunately, the measurement of the cell wall thickness is obfuscated by the thickness of the specimen. As such, the resulting binarized image (see Fig. 2) greatly overestimates the thickness of the cell wall, and must be corrected for. As it is not practical to determine the cell wall thickness from these images, the centerlines are extracted from the binarized image, and the cell wall thickness is applied based on previous studies (Table 1). Previous research has measured the values of cell wall thickness for maize cells. Data ranged from 2 micron to 6.8 microns depending on the study, samples, and methodology. The average cell wall thickness for maize as reported by several studies was found to be 4.3 microns (Table 1).

**Table 1:** Previously published cell wall thickness data based on [27–30]

Source	Nominal (um)	Tolerance (um)	Minimum (um)	Maximum (um)
Tsalagkas, 2021	3.7	1.1	2.6	4.8
Usta, 1992	6.8	N/A	6.8	***6.8***
Garay, 2009	2.0	N/A	***2.0***	2.0
Kiaei, 2011	4.6	0.9	3.6	5.6
Average	***4.3***			

Based on the values in Table 1, each model was analyzed at the average cell wall thickness value of 4.3 microns. In addition, the effect of cell wall thickness on the structural stiffness of the model was calculated, and recommendations were made for future studies. It should be noted that although the average cell wall thickness value was applied to the entire model, the process was designed so that future researchers could apply different cell wall thicknesses to each cell wall, allowing for greater detail in the analysis than is presented in this paper.

### Cell wall sensitivity study

A sensitivity analysis was performed for the structural stiffness of the model based on the cell wall elastic modulus and the cell wall thickness. The structural stiffness of the model was measured by extracting the reaction force required to stretch the right side of each model by 1 μm in the positive horizontal direction. This loading regime represents a computationally simulated standard tension test used to measure material properties. The models were then modified by either increasing the cell wall thickness by 5% or increasing the cell wall’s elastic modulus by 5%, and then reanalyzing the structure. In this way, linear normalized sensitivities about the baseline model were calculated.

## Results

### Cell segmentation

**Fig. 6:**
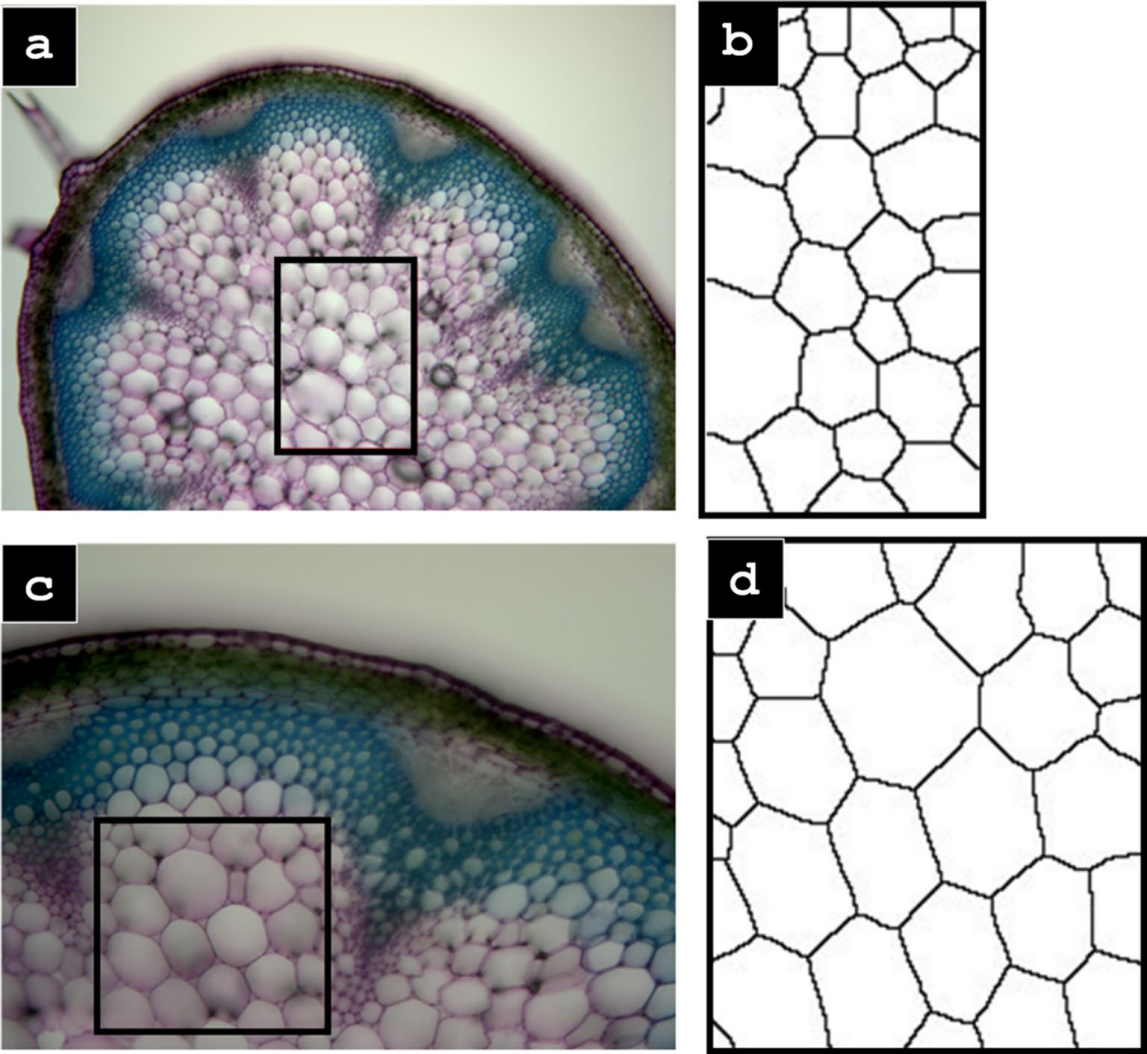
Original microscope images (top) and the resulting segmentation overlaid on the images (bottom); from left to right: *Arrabidacea verugosa*, *Mussatia hyacinthine*, *Arabidopsis thaliana*

Segmentation models of the cellular structure of *Zea mays*, *Arrabidacea verugosa, Mussatia hyacinthine, Arabidopsis thaliana* were successfully developed. To ensure the accuracy of the image processing, the resulting models were overlaid onto the original microscope images and inspected. Results were generally favorable using the WEKA segmentation method, and accurately captured the majority of the cellular structure. However, some notable shortcomings are present. First, the cells around the edges of the images tended to be less consistently modeled than the cells in towards the center of the image. Second, solitary small cells (cells that are less than approximately 1/3 of the average cell size and not surrounded by similarly-sized cells) are sometimes excluded from the segmentation, and are “shared” by their larger neighboring cells. Finally, the cellular structure is generally simplified, resulting in some loss of curvature of cell walls as well as the removal of small gaps between cells. As an example, Fig. 6 depicts the segmented splines overlaid onto the original microscope image for a sample of *Arrabidacea verugosa, Mussatia hyacinthine, Arabidopsis thaliana*.

### Finite element analyses

Finite element models were successfully developed from the extracted geometry and analyzed for stress and stiffness. Table 2 presents the resulting normalized sensitivities. The material sensitivity was similar across species, ranging from 0.96 to 1.00. The cell wall sensitivity varied from 2.3 (*Mussatia hyacinthine*) to 3.1 (*Zea mays*). Across the species investigated, it was found that the elastic modulus of the cell wall was found to have a 1.0 normalized sensitivity to structural stiffness. This means that a 5% increase in the cell wall’s elastic modulus would result in a 5% increase in the elastic modulus of the tissue the cells comprise. Conversely, the cell wall thickness was found to have a 3.0 normalized sensitivity to structural stiffness. This means that a 5% increase in the cell wall thickness would result in a 15% increase to the elastic modulus of the tissue the cells comprise.

**Table 2:** Results table from a parametric study of cell wall thickness and cell wall material stiffness

Demographics		Normalized sensitivity	
Sample #	Plant species	Material stiffness	Wall thickness
1	*Zea mays*	0.96	3.13
2	*Zea mays*	1.00	3.08
3	*Zea mays*	1.00	3.04
4	*Zea mays*	1.00	3.04
5	*Zea mays*	1.00	3.08
6	*Mussatia hyacinthine*	1.00	2.28
7	*Arabidopsis thaliana*	1.00	3.07
8	*Arabidopsis thaliana*	1.00	2.92
9	*Arrabidacea verugosa*	1.00	2.92

### Stress analysis

**Fig. 7:**
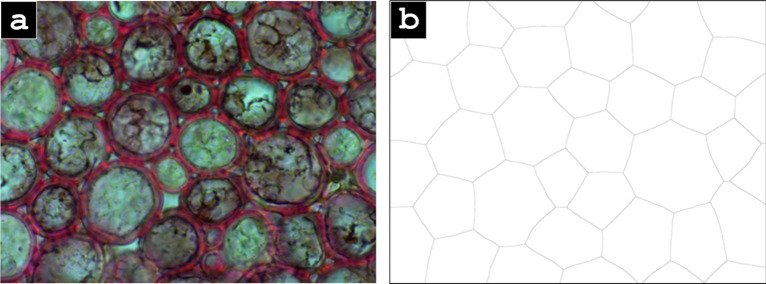
An example finite element analysis of stresses in *Zea mays* stem (100 ×), showing in cross-section the cell wall von Mises stresses from minimum (blue) to maximum (red) overlaid onto the original microscope image

Each of these models was analyzed for stress. This process allows researchers to analyze the stress in the cell walls, and then directly overlay these stresses on the original image taken from their microscope. This functionality is intended to help build intuition about the relationship between the cellular microstructure and the distribution of stresses within the cellular structure. See Fig. 7 showing an example finite element analysis of stresses in *Zea mays* stem, showing the cell wall von Mises stresses from minimum (blue) to maximum (red) overlaid onto the original microscope image.

### Other samples

Both the WEKA segmentation and the manual segmentation methods have been proven to work on images of a wide range of quality. The clarity and the resolution of the images dictates the method of segmentation as well as the ease of processing.

Figure 4 exhibits the processing steps for *Mussatia hyacinthine* stem cross-section, traditionally prepared sample. The machine learning WEKA segmentation method was used to process the image. The method was largely unchanged from the description in the WEKA section of this paper. Minor adjustments were made regarding the post-WEKA thresholding values and the post-WEKA image clean up. As demonstrated in Fig. 2, it is evident that this method can be used in both high resolution specimens prepared under the traditional method as well as the low quality image of maize used for this experiment.

The next set of images, Fig. 5 show the WEKA segmentation process being utilized on samples of *Arabidopsis thaliana* stem cross-sections. The WEKA segmentation worked on both samples; due to the clarity and resolution of the images, manual thresholding was not needed. See Fig. 5.

## Discussion

The *Arabidopsis thaliana, Mussatia hyacinthine, and Arrabidacea verugosa* images were all segmented using the WEKA method. However, for the *Zea mays* that were sectioned using the methods described in [6], manual thresholding was required as described herein. This indicates that the WEKA method is most likely sufficient for samples prepared using traditional sectioning and microscopy techniques, while additional manual work may be required to develop computational models from sections taken using the methods described in [6]. However, in either case, the resulting computational model is of comparable accuracy regardless of the method used.

Cell shape and morphogenesis are defined in higher plants by a rigid and yet still flexible cell wall [31]. The cell wall constraints the internal turgor pressure of the cell to create a myriad of shapes and structures. These structures in turn display functional importance related the ability of the organism to withstand environmental stresses. One of the major stresses impacting agricultural efficiency is lodging of the stem and data provided herein support a systematic approach to fuse biology with engineering principles surrounding the complexity of cell wall integrity.

Results of this study demonstrates the feasibility and utility of a high-throughput digitization and analysis methodology. As the models developed are fully parameterizable, detailed sensitivity studies can be performed that modify the microstructure and calculate the resulting stress state. In this way, active designing of the microstructure can be accomplished, and a microstructure ideotype can be designed and incorporated into breeding programs. Future studies can investigate further cellular and microstructural phenotypes. Specifically, the (1) organization of vertices, (2) size of the cells, and (3) curvature of the cell walls have the potential to drastically impact the stress state of the cellular structure.

Such phenotypic development is important to stalk lodging for three key reasons. First, the parametric investigation of these models can directly lead to more targeted phenotyping methods and protocols by better understanding the exact microstructural feature that needs to be measured. Second, although structural failure is often observed at the macroscopic tissue or plant level, the underlying failure mechanisms are a microscopic; cell-level material fracture and buckling directly propagate into macroscopic structural failure. Thus, through the detailed parametric analysis of the microstructure, a better understanding of the causes of structural failure can be understood. Finally, the digitization process allows for more quantitative phenotypes that can be analyzed statistically in genetic and breeding studies. Phenotypes such as cell size, cell wall orientation, etc. can be quickly quantified and analyzed using the splines output in this method.

### Limitations

The largest limitation of this study is that the cell wall digitization, quantification, and analysis is of two-dimensional images. As such, the impact of cell wall depth and the quantification of the microstructure in the third dimension is not possible. Future studies are required to expand this technique to three-dimensional cellular structures, such as the data taken from confocal microscopy of micro computed tomography scans. However, this study represents an important first step in that direction, laying the required technological foundation for such a process.

It is important to note that the development of 2-dimensional finite element models to investigate a 3-dimensional mechanism is a significant simplification. The inherent assumptions in the use of plane stress 2-dimensional models such as the ones developed herein are that (1) the top and bottom cell walls (the cell “end caps”) are negligible in stiffness, (2) the mechanical properties obtain from these models are isotropic at the tissue-level, (3) the variation in cell wall thickness is negligible, (4) the stress concentrations caused by such variations in cell wall thickness or partial thinning is ignored, and (5) the cells are hollow. All of these assumptions are most certainly incorrect, and represent significant limitations of the presented models. However, these models are a first step to be built from, with each future iteration of this technology to address these shortcomings in turn.

Additionally, there are limitations for both methods of segmentation and image processing. Neither method is 100% accurate for every image and these limits must be accounted for while processing sections of cells.

The WEKA machine learning method works best with images of cells that have less noise and clearer cell interiors. With more noise inside of the cells, the ML often confuses the inner cell with the cell walls and either misinterprets the size of the cell or excludes the cell entirely. Another limiting factor is the contrast of the cell images. Having a larger contrast will help the ML distinguish between inner cell and cell wall. Less contrast between the two categories can result in a longer processing time and potentially more error in the segmentation. The final major limit to the ML method is that it requires some user intuition on determining which cells to use as selections of the segmentation categories. The selections should include both clear and convoluted inner cells. Without a wide range of quality in the inner cell selections, the ML may only segment the cleanest of cells as the inner cells while excluding the more convoluted inner cells. Of course these limitations can be avoided based on the image contrast, brightness, and overall quality.

The manual segmentation technique has two major limits. The first is that this method requires much more user intuition since thresholding is done by the user more frequently. The second limit is that this method also requires more manual work of cleaning the cell images and removing noise. The irregularities and noise that occur in the image after thresholding must be inverted manually before creating the distance map and the Voronoi splines.

Voronoi splines are limited to linear or mostly linear cell walls. In images with cells that have highly curved walls, the Voronoi splines create a linearized shape of the cell walls. This will have adverse effects on the simulation results and will cause them to be less accurate. However, other functions will continue to work such as the automated cell counting.

**Fig. 8:**
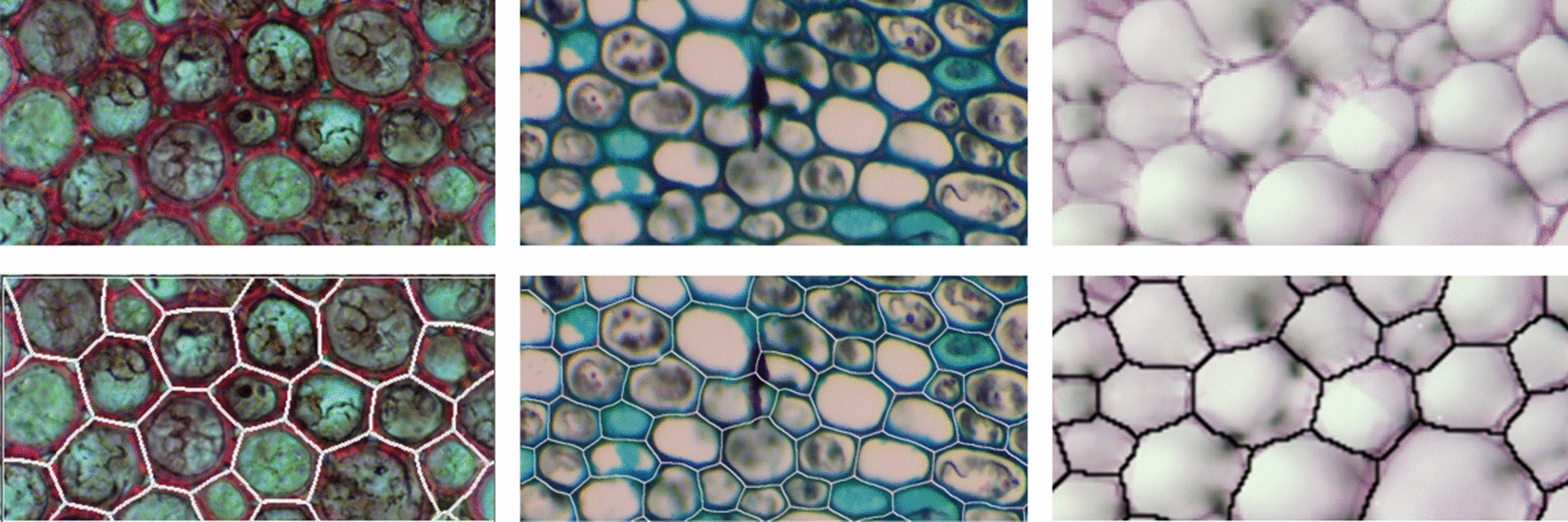
**a** *Arrabidacea verugosa* stem cross-section image (100 ×) and (**b**) the resulting Voronoi splines, which are unable to capture the highly circular cell morphology

In addition, the process does not work well for highly rounded cells, such as those of the cork cambium of *Arrabidacea verugosa* as seen in Fig. 8. However, although the resulting splines and microstructural analysis functions are inaccurate, the cell counting still functions properly.

Finally, the MATLAB algorithm was developed to separate cell walls at their vertices according to their pixel connectivity and subsequently enable the creation of splines in Abaqus FEA. However, this process had some limitations due to the difference in pixel connectivity of cell walls in segmented images. Hence, some manual input was required to correct created splines in Abaqus FEA which ensured models were adequately built for subsequent analysis.

The stated purpose of this study was to present a relatively inexpensive method for high-throughput digitization and quantification of plant cells that can be used to create two-dimensional FEMs. This is indeed a first step in a much longer process of creating 3-dimensional, full cross-section plant cell models. Thus, the sizes of the samples, though varied, are admittedly small (approximately 30–50 cells on average). This was because the models presented herein are intended to provide a demonstrable proof-of-concept methodology to build on for 3-dimensional models.

## Conclusions

Stress testing and analysis on structural members is essential to understanding the physical limitations of the morphology of plant stalks and their resistance to lodging. A robust and effective method of segmenting cell images of varied quality and creating finite element models has been presented. This method can be applied to many different images of both conventional and unconventional sectioning and staining processes. With this process, cost-effective and repeatable simulations can be conducted to aid in the understanding of stalk lodging in various crops. Readily available data from this process includes a stress analysis of cell walls, numbers of cells in an image, and degree of curvature of cell walls. Using this process in further studies could aid in the strengthening of crop stalks to prevent lodging by leveraging parametric cell wall models to develop targeted phenotypes for breeding studies.

## Supplementary Information

**Additional file 1.** Matlab code for processing voronoi splines.

## Data Availability

The Matlab functions supporting the conclusions of this article are available in the *PlantCellWallFEM* repository in https://github.com/cstubbsFDU/PlantCellWallFEM. All other datasets used and/or analyzed during the current study are available from the corresponding author on reasonable request.
